# SARS-CoV-2-Specific IgG and IgA response in maternal blood and breastmilk of vaccinated naïve and convalescent lactating participants

**DOI:** 10.3389/fimmu.2022.909995

**Published:** 2022-10-03

**Authors:** Yesica Longueira, Diego S. Ojeda, Rocio B. Antivero Battistelli, Lautaro Sanchez, Santiago Oviedo Rouco, Daniel Albano, Eleonora Guevara, Vanesa Valls, María A. Pando, Andrea V. Gamarnik

**Affiliations:** ^1^ CONICET – Universidad de Buenos Aires, Instituto de Investigaciones Biomédicas en Retrovirus y SIDA (INBIRS), Buenos Aires, Argentina; ^2^ Laboratorio de Virología Molecular, Fundación Instituto Leloir-CONICET, Buenos Aires, Argentina; ^3^ Universidad de Buenos Aires, Facultad de Medicina, Departamento de Microbiología, Parasitología e Inmunología, Buenos Aires, Argentina; ^4^ Banco de Leche Humana – Hospital Materno Infantil Ramón Sardá, Ciudad Autónoma de Buenos Aires, Argentina

**Keywords:** SARS-CoV-2, breastmilk, COVID-19 vaccine, immune response, Sputnik V, BBIBP-CorV, ChAdOx1-S

## Abstract

**Background:**

Recent studies have shown the presence of SARS-CoV-2-specific antibodies in the milk of breastfeeding mothers vaccinated with mRNA and convalescent. However, limited information is available in lactating women receiving other vaccine platforms used in developing countries, such as the inactivated SARS-CoV-2 vaccine BBIBP-CorV (Sinopharm) and the non-replicating adenovirus vaccines Sputnik V (Gamaleya Institute) and ChAdOx1-S (Oxford AstraZeneca).

**Methods:**

Here, we evaluated anti-SARS-CoV-2 IgG and IgA levels in both serum and milk samples using a longitudinal and a cross-sectional cohort of 208 breastfeeding vaccinated women from Argentina with or without previous SARS-CoV-2 infection.

**Results:**

The analysis showed that IgA levels remain constant in serum and milk of breastfeeding mothers between the first and second doses of vector-based vaccines (Sputnik V and ChAdOx1-S). After the second dose, anti-spike IgA was found positive in 100% of the serum samples and in 66% of breastmilk samples. In addition, no significant differences in milk IgA levels were observed in participants receiving BBIBP-CorV, Sputnik V or ChAdOx1-S. IgG levels in milk increased after the second dose of vector-based vaccines. Paired longitudinal samples taken at 45 and 120 days after the second dose showed a decrease in milk IgG levels over time. Study of IgA levels in serum and milk of vaccinated naïve of infection and vaccinated-convalescent breastfeeding participants showed significantly higher levels in vaccinated-convalescent than in participants without previous infection.

**Conclusion:**

This study is relevant to understand the protection against SARS-CoV-2 by passive immunity in newborns and children who are not yet eligible to receive vaccination.

## Introduction

The World Health Organization recommends exclusive breastfeeding for six months and to continue breastfeeding for two years or more due to the great benefits on babies’ and mothers’ health (1). Along with the transfer of enough nutrients to satisfy growth requirements during the first months, human milk contains both adaptive and innate immune components. In particular, breast milk immunoglobulins are essential players during the maturation of the newborn’s immune system and provide protection against pathogens (2). Research studies have shown a high concentration of immunoglobulins in breast milk also during prolonged lactation (4 years) (3). Human milk antibodies are derived primarily from B cells primed in the mucosa, resulting in high concentrations of secretory antibodies that offer a prolonged period of immune transfer to confer immunity against mucosal pathogens such as respiratory syncytial virus, pneumococcus, influenza, and meningococcus (4, 5). In particular, IgA is the dominant antibody that is transferred to infants through breast milk and is thought to play a critical role in mucosal defense (6, 7).

During the global spread of Severe Acute Respiratory Syndrome coronavirus 2 (SARS-CoV-2), the causative agent of coronavirus disease 2019 (COVID-19), studies have shown that milk produced by infected mothers contains detectable levels of anti-SARS-CoV-2 IgA and IgG during and after acute infection (8–12).The presence of these specific antibodies potentially provides passive immunization to the infant (13, 14). However, SARS-CoV-2 infection during pregnancy was associated with an increased risk of a composite outcome of maternal mortality or serious morbidity from obstetric complications (15). This highlights the importance of vaccination, since vaccines induce a strong antibodies production by pregnant women.

Although vaccination against COVID-19 is the most effective way to prevent SARS-CoV-2 infection and transmission, pregnant and breastfeeding women were not included in the original vaccine trials. However, as this group has been associated with high rates of preterm birth and neonatal morbidity (16, 17), pregnant and lactating women were included in subsequent vaccination trials. Recommendations to prioritize these groups are supported by the effectiveness (18, 19) and safety (20–23) of different COVID-19 vaccines. Several studies evaluated the presence of anti-SARS-CoV-2 IgA and IgG in the breast milk of lactating mothers vaccinated with mRNA and non-replicating adenovirus vaccines (24–30), however, scarce information is available with inactivated virus platforms widely used in many regions of the world (31).

Different anti-SARS-CoV-2 vaccines are currently used in Argentina, including the non-replicating adenovirus vaccines Sputnik V (Gamaleya Institute), ChAdOx1-S (Oxford AstraZeneca), and Ad5-nCoV (CanSino); the mRNA vaccines BNT162b2 (Pfizer) and mRNA-1273 (Moderna); and the inactivated SARS-CoV-2 vaccine BBIBP-CorV (Sinopharm). As of today, August 2022, 91% of the total population have received at last one dose of the COVID-19 vaccine, 84% have received two doses and 60% have received three or four doses. The national vaccination plan included the entire population from 3 years of age. Unfortunately, stratified data on vaccination coverage by age is not available (32). Due to the lack of information regarding immunogenicity in breast milk in lactating women after the application of vaccine platforms based on viral vectors (Sputnik V and ChAdOx1-S) or inactivated viruses (BBIBP-CorV), we evaluated the presence of specific IgA and IgG anti-SARS-CoV-2 in maternal blood and breast milk of vaccinated lactating participants without prior infection and convalescent lactating participants who were vaccinated with diverse vaccine platforms.

## Material and methods

### Population

Breastfeeding mothers from the Human Milk Bank (HMB) at the Hospital Materno-Infantil Ramón Sardá were donors in this study. We expanded this cohort with volunteers outside the HMB, including breastfeeding women that were enrolled by social network advertisements. Serum and breast milk samples were obtained from lactating mothers before and after vaccination against SARS-CoV-2. From February 2021 to February 2022, samples were taken from 226 breastfeeding women from Buenos Aires City and surroundings. During the study period, vaccination for COVID-19 advanced substantially in Argentina, registering two waves of contagion (May-June 2021 and January 2022). Of this, 171 naïve participants received one or two doses of the vaccines available at that time in Argentina (Sputnik V, ChAdOx1‐S or BBIBP‐CorV). None of these vaccinated mothers reported clinical COVID-19 infection before immunization. Participants were also separated as naïve or convalescent by measuring the presence of anti-nucleocapsid antibodies. Additionally, we evaluated the National COVID-19 Surveillance System, which includes many asymptomatic cases tested during surveillance and as close contacts of symptomatic cases, to identify if there were previously infected volunteers (33).

**Figure 1:**
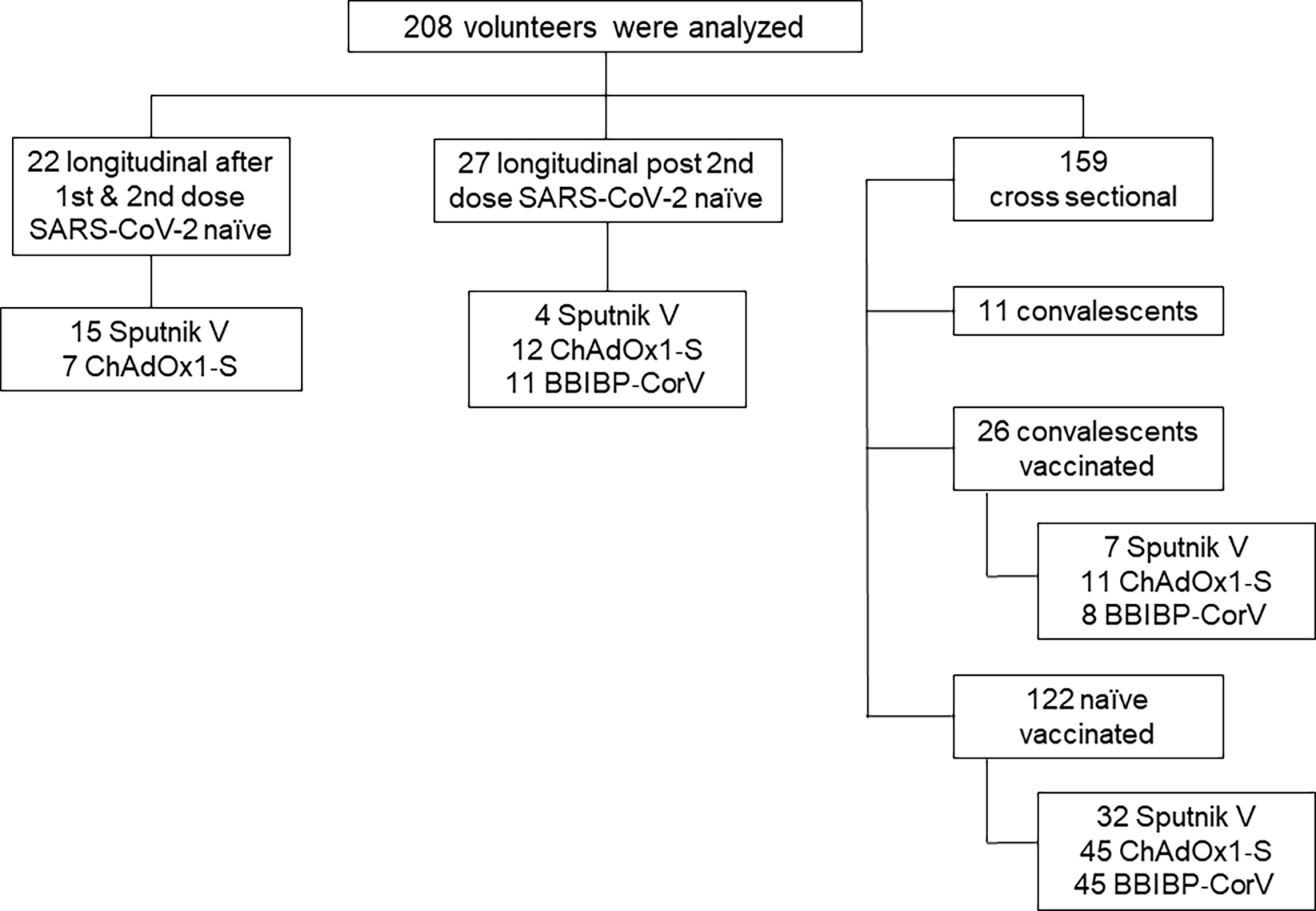
Flowchart of the study cohort. For longitudinal analysis, the geometric mean time interval for the samples obtained was 65 and 21 days after first and second doses of Sputnik V and ChAdOx1‐S vaccines.

For this longitudinal and cross-sectional study, 208 breastfeeding mothers’ serum and breast milk samples were analyzed. We evaluated three data sets. Al first, we analyzed 44 paired serum and breast milk samples from 22 vaccinated breastfeeding mothers without prior SARS-CoV-2 infection. In this case, the samples were obtained longitudinally after the first and second dose of Sputnik V and ChAdOx1‐S vaccine application. Then, we analyzed a longitudinal cohort of 27 naïve vaccinated mothers with two doses of Sputnik, ChAdOx1‐S or BBIBP-CorV vaccines. These samples were collected as a function of time after the second dose, at 40 and 120-day after completing the vaccination schedule. Finally, the third group was a cross sectional cohort composed of 122 vaccinated mothers without previous SARS-CoV-2 infection (Sputnik V, N=32, ChAdOx1‐S, N=45 and BBIBP-CorV N=45), in addition this group included 26 vaccinated convalescents, with SARS-CoV-2 infection confirmed by molecular diagnosis before vaccination and 11 convalescents non vaccinated breastfeeding mothers (**Figure 1**).

### Clinical data collection

Inclusion criteria included women ≥ 18 years of age who were breastfeeding at any infant age. Data collected included age of mother and infant, vaccine type (Sputnik V, ChAdOx1-S and BIBP‐CorV), vaccination dates and history of SARS-CoV-2 infection. Ethical approval was obtained from the Institutional Review Board (IRB) of the Faculty of Medicine of Buenos Aires University (Comité de Ética en Investigación Biomédica, Instituto Alberto C. Taquini de Investigaciones en Medicina Traslacional (IATIMET), Facultad de Medicina, Universidad de Buenos Aires). Informed consent was obtained from all study participants.

### Sample collection and processing

Mothers were virtually instructed by study staff in clean techniques to obtain milk samples. Women collected the milk in sterile containers that were immediately frozen until shipment in a cooler to INBIRS (Instituto de Investigaciones Biomédicas en Retrovirus y Sida). Once at the laboratory, human milk samples were stored at −20 ◦C until use. The volunteers assisted to the laboratory were blood was drawn.

Samples consisted of 15 mL of milk and 5 mL of venous blood without anticoagulants. Both types of samples were collected on the same day. Blood was centrifuged at 2500 revolutions per minute (rpm) for 10 min at room temperature, and sera were aliquoted in cryogenic vials and stored at −20 ◦C until use. Prior to processing, breast milk samples were thawed centrifuged at 1500 rpm for 15 min, fat was removed, and supernatant was transferred to a new tube. Centrifugation was repeated 2× to ensure removal of all cells and fat, and the supernatant was aliquoted into cryogenic vials and stored at −20 ◦C until use. All serum and breast milk samples were tested in parallel on two different SARS-CoV-2 antibodies testing platforms, which are described in detail below. Evaluation of possible previous asymptomatic SARS-CoV-2 infection was assessed by measuring the presence of IgG anti nucleocapsid by ELISA.

### Detection of specific SARS-CoV-2- antibodies in serum and breast milk

Antibodies to SARS-CoV-2 spike protein were detected using an established commercially available two-step ELISA (COVIDAR) for IgG in serum samples. We have previously described the development of the ELISA for IgG in serum samples (34). Modifications of the ELISA for IgA in serum and IgG/IgA in breast milk samples are described below. Serum and breast milk samples diluted in PBS-T containing 0.05% Tween and 0.8% casein were added to the plate (200 μl of a 1:50 dilution for IgG and IgA determination in serum and 200 μl of a 1:8 dilution for IgG and IgA in breast milk), and incubated for 1 h at 37°C for serum samples and for 2h at 24°C for breast milk samples. Following a washing step with PBS-T, 100 μl of diluted horseradish peroxidase (HRP)-conjugated with goat anti-human IgA (Sigma), or with mouse anti-human IgG antibodies (BD pharmingen), was added to plates and incubated for 30 min at 37°C for serum, or 1 h at 37°C for milk. The conjugated monoclonal antibody used for human IgG detection in the COVIDAR ELISA is G18-145, which specifically binds to the heavy chain of all four human immunoglobulin G subclasses: IgG1, IgG2, IgG3, and IgG4. The conjugate employed for IgA detection in human specifically binds to α-chain specific of human immunoglobulin A (SIGMA, Cat A0295-1ML). Subsequently, the plates were washed with PBS-T, and the peroxidase reaction was visualized by incubating the plates with 100 μl of TMB solution for 30 min. at 37°C for serum samples and for 1 h at 24°C in breast milk samples. The reaction was stopped by adding 100 μl of 1M sulfuric acid, and optical densities (OD) were immediately measured at 450 nm. Cut-off for serum and breast milk samples resulted from the mean of OD450 values from negative controls plus 3 times the standard deviation. All the assays, IgG and IgA determinations in serum and breast milk samples, were performed simultaneously with the same plate batch. The IgG concentration of serum sample, expressed in international units per milliliter (BAU/ml), was calculated by extrapolation of the optical density at 450 nm (OD450) on a calibration curve built using serial dilutions of the WHO International Standard for anti-SARS-CoV-2 immunoglobulin.

IgG antibodies against SARS-CoV-2 nucleocapsid protein were detected using a in house two-step ELISA test. The assay uses plates coated with 100 ng of the full-length nucleocapsid protein, expressed in E. Coli and purified using HisTrap excel columns and the conjugated monoclonal antibody was the same as the one used for COVIDAR. The assay was validated using a panel of 170 healthy blood donors obtained pre-pandemic as negative control. The cut off was set as the mean of negative control plus 3 standard deviations and was defined to maximize specificity (**Figure S1A**). We measured anti SARS-CoV-2 nucleocapsid IgG of serum samples included in this study (**Figure S1B**). Samples from individuals vaccinated with Sputnik V or ChAdOx1-S yielded negative results. In addition, convalescent, vaccinated convalescent and vaccinated with BBIBP-CorV groups yielded 100, 92 and 88% positive results, respectively (see **Figure S1**).

### Quantification and statistical analysis

**Figure 2:**
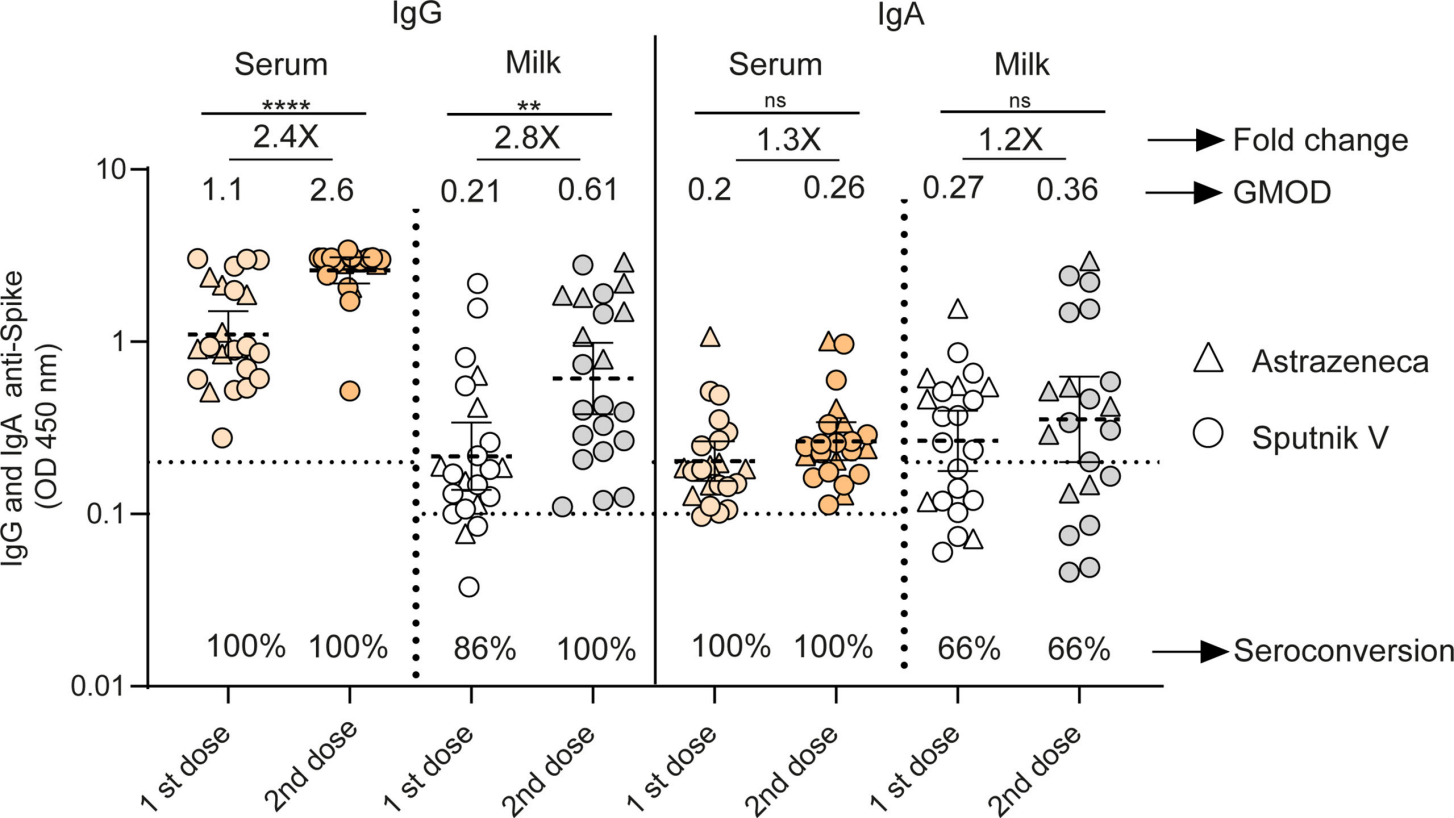
Longitudinal antibodies measurements between the 1st and 2nd doses of adenoviral-based vaccines in serum and breast milk of lactating women. Anti-spike IgG and IgA antibody levels measured as geometric means by OD at 450 nm receiving Sputnik V C1 and C2 vaccine (Gamaleya, N= 15) and ChAdOx1-S vaccine (AstraZeneca, N= 7). Cut-off for serum and breast milk samples resulted from the mean of OD450 values from negative controls plus 3 times the standard deviation and is shown as dotted line. Samples were obtained as a function of time at 65 (95%CI: 54 to 79 days) and 21 (95%CI: 18 to 26 days) days after first and second dose of Sputnik V and ChAdOx1‐S vaccines. Wilcoxon matched-pair test was used. Statistical significance is shown with the following notations: ****, P < 0.0001; **, P < 0.01; ns, not significant.

**Figure 3:**
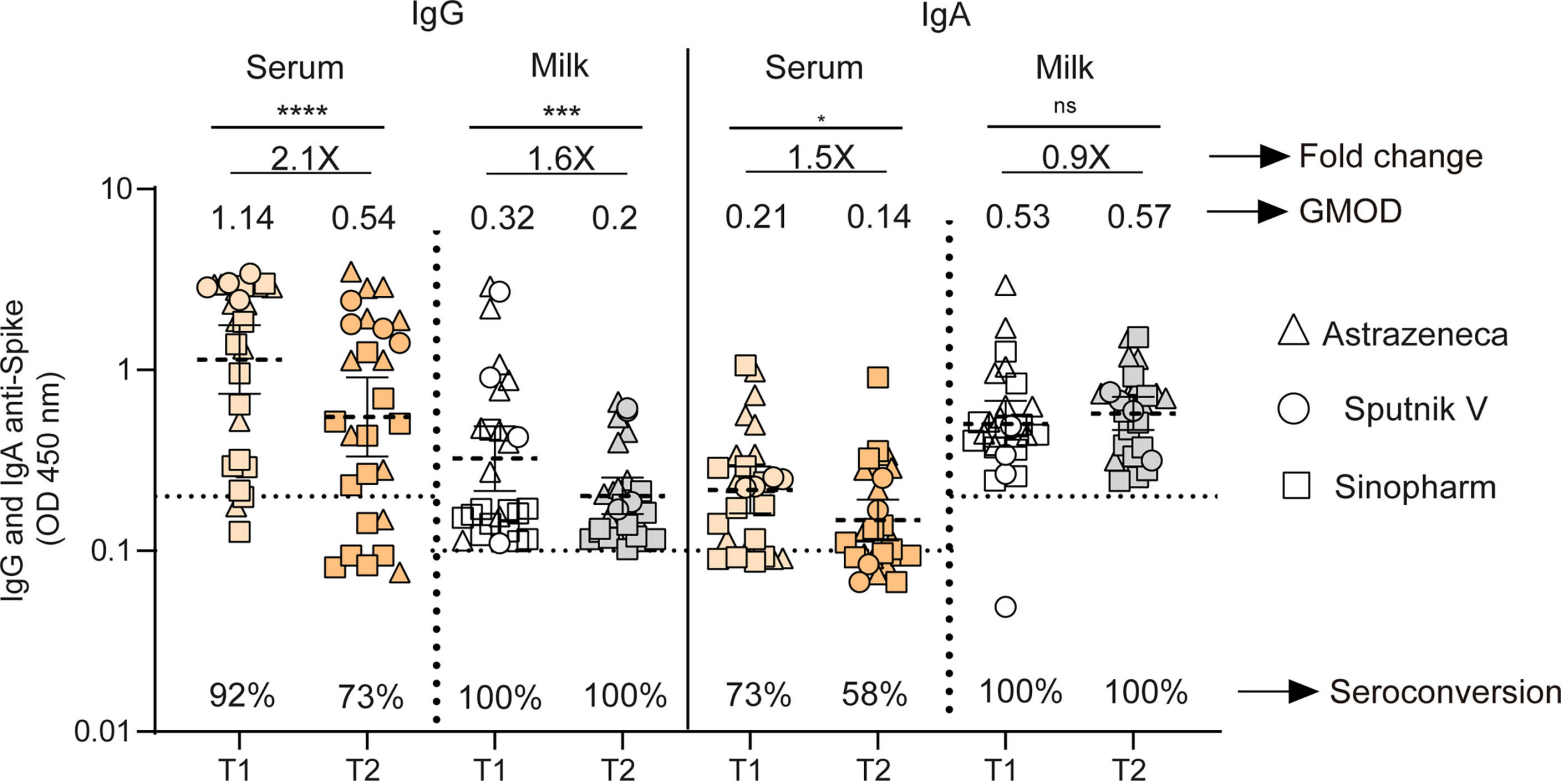
Longitudinal antibodies measurements in fully vaccinated breastfeeding without previous infection. IgG and IgA anti-spike antibody levels measured as geometric means by OD at 450 nm in naïve mothers receiving Sputnik V C1 and C2 vaccine (Gamaleya, N= 4), ChAdOx1-S vaccine (AstraZeneca, N= 12) and BBIBP10 CorV vaccine (Sinopharm, N=12). Samples were obtained as a function of time at 44 (T1) and 120 (T2) days after second doses of Sputnik V, ChAdOx1‐S and BIBP-CorV vaccines. Cut-off for serum and breast milk samples resulted from the mean of OD450 values from negative controls plus 3 times the standard deviation and is shown as dotted line. Wilcoxon matched-pair test was used. Statistical significance is shown with the following notations: ****, P < 0.0001; ***, P < 0.001; *, P < 0.05; ns, not significant.

**Figure 4:**
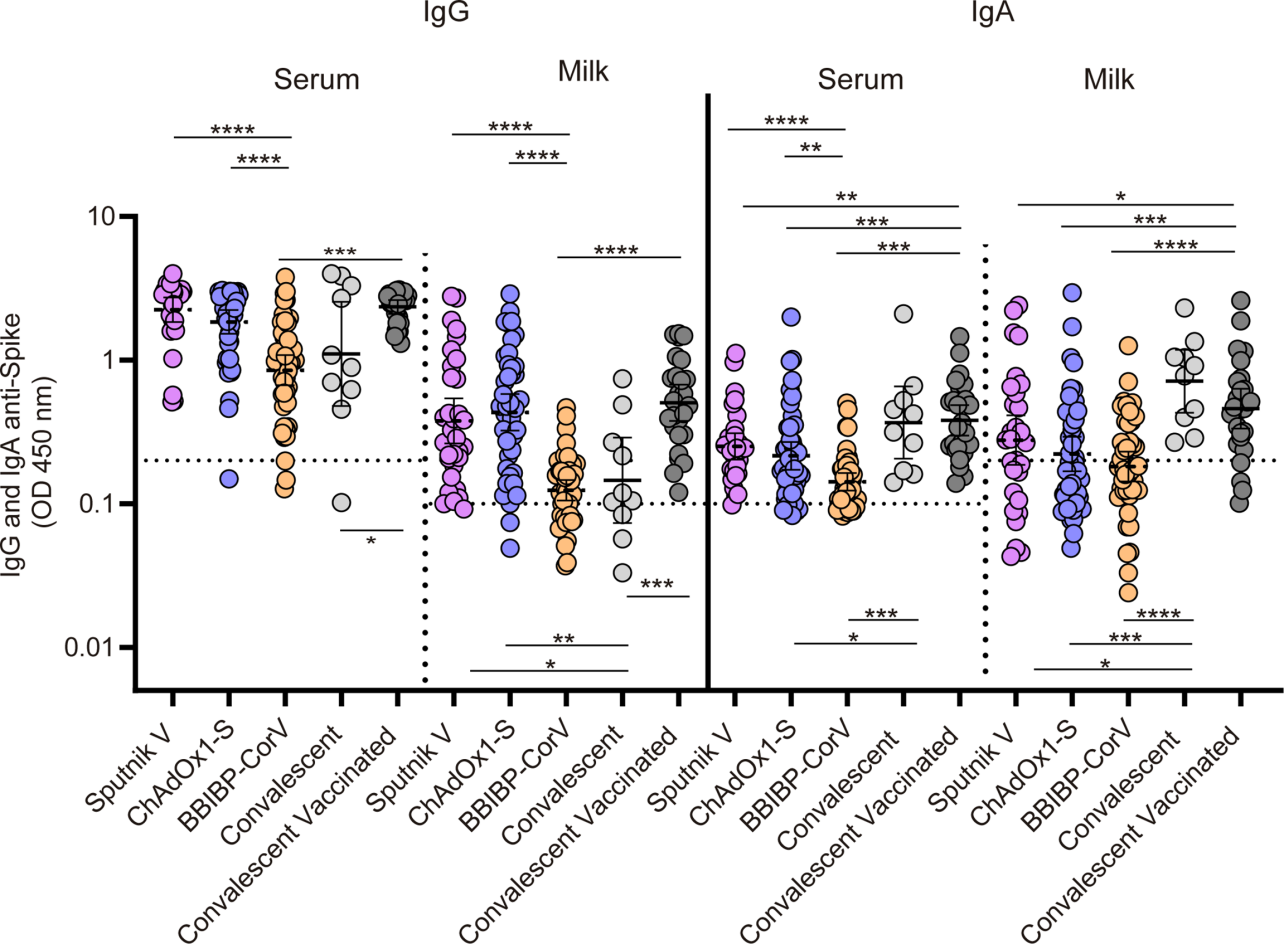
Cross sectional analysis of antibody responses in serum and breast milk in vaccinated naïve, vaccinated convalescent and convalescent breastfeeding women. IgG and IgA anti-spike antibody levels measured as geometric means by OD at 450 nm. Cut-off for serum and breast milk samples resulted from the mean of OD450 values from negative controls plus 3 times the standard deviation and is shown as dotted line. Samples were obtained at 30 (95%CI: 23 to 47 days), 35 (95%CI: 29 to 42 days) and 52 (95%CI: 45 to 62 days) days after second dose in mothers without previous SARS-CoV-2 infection vaccinated with Sputnik V, ChAdOx1‐Sand BIBP-CorV respectively. In addition, samples from convalescents vaccinated and convalescents non vaccinated breastfeeding mothers were obtained at 36 (95%CI: 29 to 46 days) days after second dose and at 163 (95%CI: 93 to 285 days) days after symptoms onset. For no paired samples analysis, Kruskal-Wallis One-Way ANOVA was performed to compare antibody response. Statistical significance is shown with the following notations: ****, P < 0.0001; ***, P < 0.001; **, P < 0.01; *, P < 0.05.

All statistical tests and plots were performed using GraphPad Prism 8.0 software. Comparisons of antibody concentration were made using two-tailed Wilcoxon matched-pair test in **Figures 2**, **3**. Comparison on non-paired determinations of antibody concentration was made using the One Way ANOVA Krustal-Wallis test in **Figure 4**. Statistical significance is shown in the figure legends with the following notations: ****, P < 0.0001; ***, P < 0.001; **, P < 0.01; *, P < 0.05; ns, not significant. Geometric means with 95% confidence intervals were calculated for **Figures 2**–**4**. Spearman correlation coefficient was used to calculate correlations between serum and human breast milk IgG and IgA. A two-tailed p-value lower than 0.05 was considered as significant.

## Results

### IgA levels are constant between the 1^st^ and 2^nd^ doses of adenoviral-based vaccines in serum and breast milk of lactating women

We evaluated SARS-CoV-2-specific IgG and IgA responses in human serum and breast milk paired samples of 22 volunteers without prior SARS-CoV-2 infection after one and two doses of Sputnik V (N=15) or after one or two doses of ChAdOx1‐S (N=7). Application of the second dose increased the IgG level in both serum and breast milk samples (p<0.0001 and p<0.001 respectively) (**Figure 2**). In serum, the IgG levels (measured as geometric means by OD at 450 nm, GMOD) were 1.1 after the first dose, and 2.6 after the second dose (95% confidence interval [CI], 0.8 to 1.5 and 2.1 to 3, respectively); and in breast milk, the levels were 0.21 after the first dose and 0.61 after the second dose (95% CI, 0.13 to 0.33 and 0.37 to 0.98, respectively). The increase in IgG was statistically significant (2.4- and 2.8-fold in serum and milk, respectively). In contrast, IgA level remained constant in serum and breast milk between the two doses (**Figure 2**). The seroconversion of IgG was 100% in serum and milk after the second dose, while the seroconversion of IgA in serum and milk was 100 and 66%, respectively.

### Vaccination in breastfeeding women is associated with sustained IgA level in milk over time

We then evaluated IgG and IgA responses across both compartments in 27 paired samples from lactating women without prior SARS-CoV-2 infection. For this group, samples were obtained as a function of time at 44 (95% CI 34 to 56 days) and 120 (95% CI 114 to 124 days) days after second doses of Sputnik V, ChAdOx1‐S and BIBP-CorV vaccines. For the vaccinated participants, the IgG level waned significantly across both serum and breast milk, showing a 2.1- and 1.6-fold decrease over time, respectively (p<0.0001 and p<0.001). IgA level declined slightly over time in serum, while it was sustained in breast milk (**Figure 3**). In addition, the seropositive rate declined in circulating IgG and IgA, while no significant changes in this rate was observed in breast milk compartments over time after the second dose (**Figure 3**).

### Antibody responses in serum and breast milk in vaccinated naïve, unvaccinated convalescent and vaccinated convalescent breastfeeding women

The IgG and IgA responses in both serum and breast milk after the second dose of Sputnik V (N=32), ChAdOx1‐S (N=45) and BIBP-CorV (N=45) vaccine application were compared with those of unvaccinated convalescent (N=11) and convalescent fully vaccinated (N=26) breastfeeding women. IgG levels in serum and milk from participants receiving the adenoviral-based vaccines reached higher levels than those observed in those vaccinated with inactivated SARS-CoV-2 Sinopharm vaccine (**Figure 4**). Specific-IgA response in breast milk showed no significant differences after vaccination with Sputnik V, ChAdOx1‐S or BIBP-CorV. The subset of vaccinated mothers with previous SARS-CoV-2 infection showed the highest IgG level in serum and milk. Finally, a robust IgA response in both serum and breast milk was evidenced in unvaccinated and vaccinated convalescent mothers, showing a significant difference compared to vaccinated naïve participants (**Figure 4**).

### Correlation of SARS CoV-2 specific IgG and IgA antibodies in paired breast milk and serum samples

**Figure 5:**
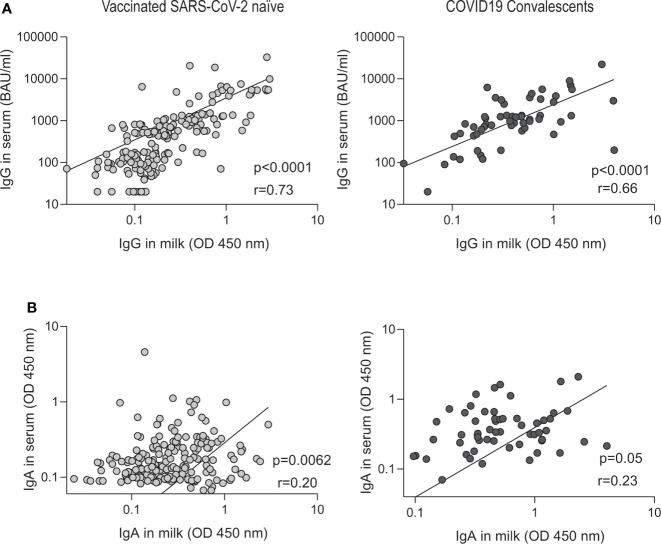
Correlation between paired breast milk and serum SARS-CoV-2-specific antibody in vaccinated naïve and convalescent breastfeeding women. Correlation of IgG (quantified according to the WHO International Antibody Standard in serum) and IgA anti-spike antibody levels measured by OD at 450 nm in vaccinated naïve (n=122) and convalescents plus vaccinated convalescents (n = 47). The specific IgG **(A)** and IgA **(B)** serum levels are correlated to the breast milk levels. In the inset the r Spearman and p values from linear regression are shown.

Comparison of paired SARS CoV-2 IgG antibodies in serum and breast milk shows high correlation in the two groups analyzed: vaccinated naïve (IgG correlation coefficient r =0.73, P < 0.0001; **Figure 5A**) and convalescents volunteers (IgG correlation coefficient r =0.66, P < 0.0001; **Figure 5A**). In contrast, low correlation was observed when specific IgAs were analyzed in vaccinated naïve volunteers (IgA correlation coefficient r =0.20, P = 0.0062; **Figure 5B**) and convalescents volunteers (IgA correlation coefficient r =0.23, P = 0.05; **Figure 5B**).

## Discussion

Comparison of SARS-CoV-2-specific antibodies in breast milk and serum after vaccination has been mainly described for mRNA and non-replicating adenovirus vaccines (24–30) used in most developed countries. However, little information was available on widely used inactivated virus platforms in many regions of the world (31). This study provides data about longitudinal antibody responses to adenoviral-based vaccines (Sputnik V and ChAdOx1‐S) and inactivated SARS-CoV-2 vaccine (BIBP-CorV) in SARS-CoV-2 naive and previously infected breastfeeding mothers.

We observed sustained levels of IgA between the first and second doses of adenoviral-based vaccines Sputnik V and ChAdOx1-S. These levels were maintained over time up to 120 days after vaccination. We also showed that the IgA response in breast milk did not show significant differences after vaccination with Sputnik V, ChAdOx1‐S or BIBP-CorV. In contrast, the adenoviral-based vaccines achieved higher IgG levels than those observed in individuals vaccinated with the inactivated BBIBP-CorV vaccine in both serum and milk. Regarding previously infected volunteers, convalescent and vaccinated convalescents lactating women showed a robust IgA response in both serum and breast milk compared to unvaccinated volunteers. Also, vaccinated mothers with previous SARS-CoV-2 infection showed the highest IgG level in serum and milk.

We observed that the IgG levels increase after the second dose of the Sputnik V and ChAdOx1‐S, with 100% of the participants showing IgG positivity in milk and serum. Similar observations were previously reported for both, mRNA and adenoviral based vaccines (27, 28). This rapid increase of IgG after the second dose is consistent with a specific B lymphocyte memory that will prime a faster response with higher antibodies levels (33). In contrast, we observed that the IgA levels remained constant between the two doses, as it was reported in previous studies with adenoviral-based vaccines (Ad26.COV2.S and ChAdOx1‐S) (27, 28, 35). Heterogeneous dynamics in IgG and IgA antibody levels can be associated to their diverse functions. IgA shows a key role dominated in the early SARS-CoV-2–specific antibody response and IgG is predominantly important in the secondary immune response (36).

Analysis of paired longitudinal samples taken at 45 and 120 days after second vaccination dose showed that, while IgG levels waned over time in milk, the IgA levels were maintained and 100% of the participants displayed IgA in milk. We detected a slight reduction of IgA titers in serum relative to paired breast milk samples obtained 120 days after the second dose of Sputnik V, ChAdOx1‐S and BBIBP-CorV vaccines, suggesting a more sustained IgA level in mucosal secretions. Previous studies observed that IgA antibody levels slightly decreased 70 days after the second dose of mRNA vaccine administration (37). It is important to mention that other studies showed a decrease in IgA after 90 days of a second dose of the inactivated SARS-CoV-2 vaccine (30). In contrast to that observed for IgA, a significant decreased was observed in IgG levels both in serum and breastmilk pared samples. These results are in agreement with previous studies using mRNA-based vaccines that showed increased IgG levels after the first and second doses, with a significant reduction thereafter (35, 37, 38).

A cross sectional study with 159 samples showed that the mean IgA levels in the milk of breastfeeding women who received three different vaccine platforms were similar. No significant differences were observed in IgA levels after the application of technologies based on vectors or viruses inactivated vaccines. When this cohort was compared to samples from convalescent or convalescent/vaccinated participants, a significant difference in milk IgA levels was observed, indicating that infection results in a higher IgA response. In agreement with previous studies, COVID-19 convalescents were associated with an elevated IgA response in human milk and these levels were higher than those observed in vaccinated groups (37, 39). Furthermore, the IgA response in milk was not significantly different when convalescents unvaccinated and convalescents vaccinated participants were compared.

Regarding the IgG response in breast milk, vaccination of infected volunteers resulted in a strong and long-term IgG response. Differences in milk IgG levels were observed with all three vaccines platforms, showing higher levels when the vector-based vaccines were used compared to the inactivated virus vaccine. Previous studies have demonstrated greater IgG levels in the serum of volunteers (general population cohort) vaccinated with adenoviral vector vaccines when compared to that with virus inactivated vaccines (40–42). However, there are no reports of antibody response comparing adenoviral vector-based and inactivated virus vaccines in paired samples of milk and serum from lactating mothers. Regarding convalescent volunteers, significantly lower levels of IgG were detected in milk and serum in unvaccinated convalescents compared to vaccinated convalescents. In this regard, previous studies also provided data showing that IgG levels in vaccinated SARS-CoV-2 naïve and vaccinated convalescents mothers were higher than those observed in unvaccinated convalescents (28, 43).

We also found a higher correlation with IgG than with IgA in breast milk and serum paired samples, which demonstrates a greater accumulation of IgA in breast milk as reported in other studies with mRNA vaccines (7). This lack of IgA correlation between serum and breast milk is in agreement with the results shown in **Figures 2**, **3** and with previous studies demonstrating that the IgA response was greater in breastmilk than in serum (44). Even more, these results are consistent with a previous work that demonstrated that IgA in breast milk is produced by plasma cells that are accumulated in the lactating mammary glands (45). These plasma cells are primed in the lymph nodes and in the Peyer’s patches of the mucosal tissues and home to the mammary glands in lactation (46). A similar Spearman´s correlation coefficient was observed for IgG in serum and milk samples from vaccinated and convalescent donors.

This study includes the largest cross-sectional analysis of human serum and breast milk samples after Sputnik V, ChAdOx1‐S and BIBP-CorV vaccination in breastfeeding women compared with unvaccinated-convalescent and vaccinated-convalescent participants. Our data add to previous information generated with mRNA vaccine platforms to support the idea that SARS-CoV-2 vaccination in lactating women provides passive immunity for the recipient infant against this virus.

## Data availability statement

The raw data supporting the conclusions of this article will be made available by the authors, without undue reservation.

## Ethics statement

The studies involving human participants were reviewed and approved by Comité de Ética en Investigación Biomédica del Instituto Alberto C. Taquini de Investigaciones en Medicina Traslacional (IATIMET), Facultad de Medicina. The patients/participants provided their written informed consent to participate in this study.

## Author contributions

AG, MP and DO are the principal investigator, designed and performed research, and coordinated the study. DA, VV and EG recruit’s mothers included in the Human Milk Bank of the Hospital Materno Infantil Ramón Sardá´s as regular donors to participated in the study. RB recruit the volunteers through social networks advertisements. YL, RB and MP coordinated the study. YL, RB and MP were involved in sample processing and collection. DO and LS were involved in ELISA protocols modifications for IgA in serum and IgG/IgA in breast milk samples. DO and SR validated ELISA anti-nucleocapsid IgG. DO and LS built of the data base. DO and LS performed the statistical analysis and interpretation of the data. YL, DO, RB and LS were involved in data collection, organization, coordination, and technical support of the study. AG, MP, YL and DO wrote and edited the manuscript. All authors had full access to all data in the studies, critically reviewed the manuscript, approved the final version and had final responsibility for the decision to submit for publication.

## Funding

This work has received funding from NIH (NIAID) R01AI095175 to AVG, Fondo Nacional para la Investigación Científica y Tecnológica de Argentina PICT 201902869 to AVG, Fundación Williams to AVG, Fondo para la Convergencia Estructural del MERCOSUR (FOCEM) and NIH U19AI168631-0 to AVG.

## Acknowledgments

The authors would like to thank to María José Quiroga and all the mothers who agreed to participate in this study, as well as, the staff of the centers where recruitment was performed. We are grateful with the members of the Gamarnik laboratory for helpful discussions and with the Lab SeVa group who contributed in sample determination in the beginning of the project.

## Conflict of interest

The authors declare that the research was conducted in the absence of any commercial or financial relationships that could be construed as a potential conflict of interest.

## Publisher’s note

All claims expressed in this article are solely those of the authors and do not necessarily represent those of their affiliated organizations, or those of the publisher, the editors and the reviewers. Any product that may be evaluated in this article, or claim that may be made by its manufacturer, is not guaranteed or endorsed by the publisher.
