# Supplementary material for: SARS-CoV-2-Specific IgG and IgA response in maternal blood and breastmilk of vaccinated naïve and convalescent lactating participants
Source: Front Immunol. 2022 Oct 3;13:909995. doi: 10.3389/fimmu.2022.909995 (PMC9574440; doi:10.3389/fimmu.2022.909995)

**Figure S1:** *ELISA anti-nucleocapsid IgG validation*. IgG anti-nucleocapsid antibody levels measured as geometric means by OD at 450 nm. A). Levels of specific anti-nucleocapsid IgG antibodies against SARS-CoV-2 in 170 pre-pandemic serum samples. The cut-off was defined as the mean of pre-pandemic samples plus 3 standard deviations and is shown as dotted line. B) Sensibility of *anti-nucleocapsid* IgG antibodies against SARS-CoV-2 in serum from lactating seronegative women, vaccinated with adenoviral vector (Sputnik and ChAdOx1-S), vaccinated with inactivated virus (BBIBP-CorV), convalescent and vaccinated convalescent.


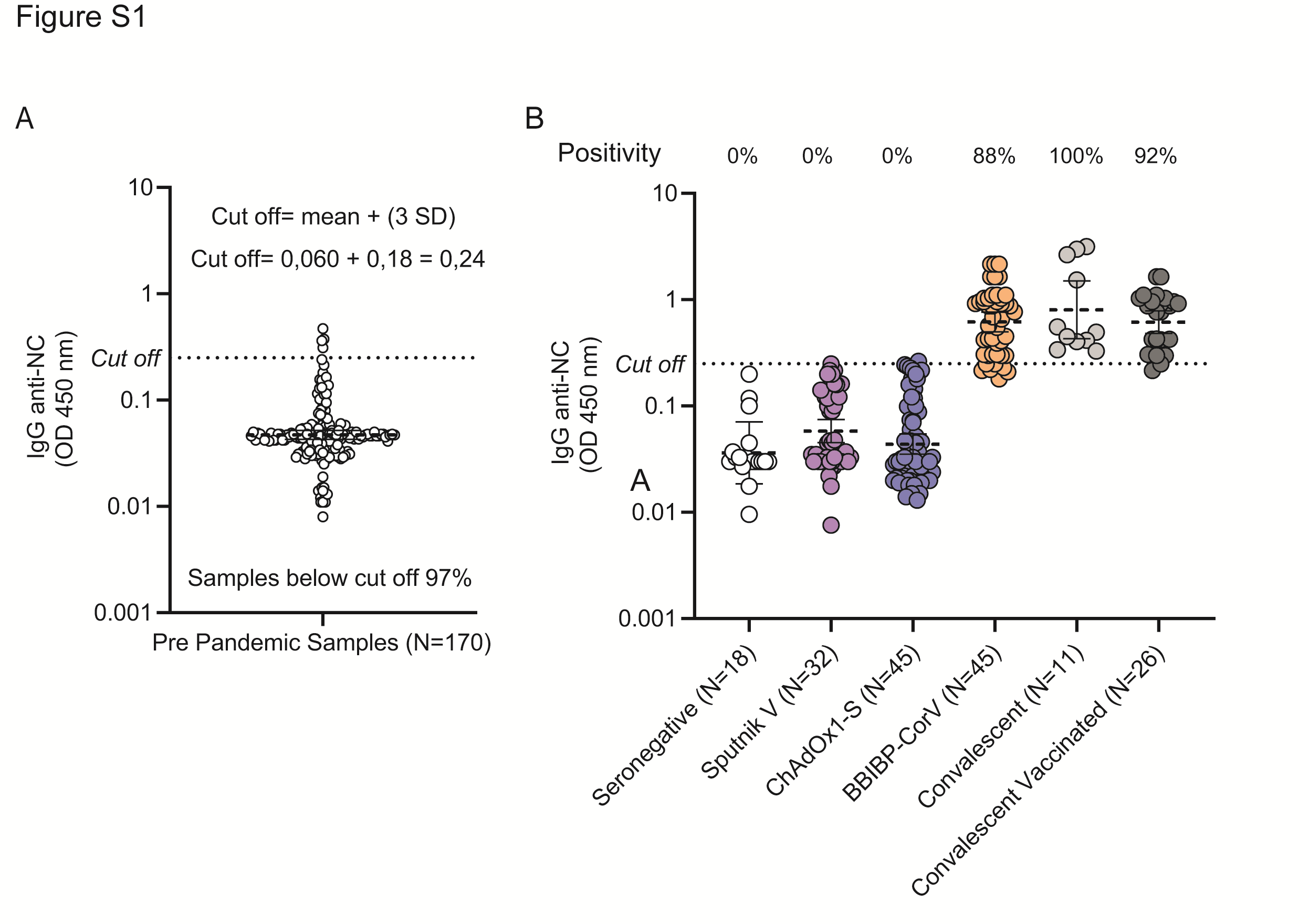

Supplement: Supplementary file 1 [file DataSheet_1.docx]
